# A survey of Italian and Spanish neonatologists and paediatricians regarding awareness of the diagnosis of FAS and FASD and maternal ethanol use during pregnancy

**DOI:** 10.1186/1471-2431-11-51

**Published:** 2011-06-06

**Authors:** F Vagnarelli, I Palmi, O García-Algar, M Falcon, L Memo, L Tarani, R Spoletini, R Pacifici, C Mortali, A Pierantozzi, S Pichini

**Affiliations:** 1 Arcispedale Santa Maria Nuova NICU, Reggio Emilia, Italy; 2 Istituto Superiore di Sanità, Rome, Italy; 3 URIE, Hospital del Mar, Institut Municipal d'Investigacio Medica (IMIM), Barcelona, Spain; 4 Deparment of Legal Medicine, Universidad de Murcia, Murcia, Spain; 5 San Martino Hospital - Paediatric Unit, Belluno, Italy; 6 Policlinico Umberto I Clinical Genetics, Roma, Italy; 7 ARS Toscana, Osservatorio Qualità, Italy

## Abstract

**Background:**

Ethanol is the most widely used drug in the world and a human teratogen whose consumption among women of childbearing age has been steadily increasing. There are no Italian or Spanish statistics on ethanol consumption during pregnancy nor any information regarding prevalence of fetal alcohol syndrome (FAS) and fetal alcohol spectrum disorders (FASD). There is also a reasonable suspicion that these two diseases are underdiagnosed by professionals from the above-reported countries. The objectives of this study were: 1) to evaluate the experience, knowledge and confidence of Italian and Spanish neonatologists and paediatricians with respect to the diagnosis of FAS and FASD, and 2) to evaluate professionals awareness of maternal drinking patterns during pregnancy.

**Methods:**

A multiple-choice anonymous questionnaire was e-mailed to Italian neonatologists registered in the mailing list of the corresponding Society and administered to Italian and Spanish paediatricians during their National Congress.

**Results:**

The response rate was 16% (63/400) for the Italian neonatologists of the National Society while a total of 152 Spanish and 41 Italian paediatricians agreed to complete the questionnaire during National Congress. Over 90% of the surveyed physicians declared that FAS is an identifiable syndrome and over 60% of them identified at least one of the most important features of FAS. Although over 60% Italian responders and around 80% Spanish responders were aware that ethanol use in pregnancy is dangerous, approximately 50% Italian responders and 40% Spanish ones allowed women to drink sometimes a glass of wine or beer during pregnancy.

Neonatologists and paediatricians rated confidence in the ability to diagnosis FAS and FASD as low, with over 50% responders feeling they needed more information regarding FAS and FASD identification in newborn and child.

**Conclusions:**

Italian and Spanish neonatologists and paediatricians do not feel confident about diagnosing FAS and FASD. More training is needed in order to accurately diagnose ethanol use during pregnancy and correctly inform pregnant women on the consequences on the newborn.

## Background

Italy and Spain are two Mediterranean countries which, due to their position in Europe, are the centre of both industrial and cultural vitality, attracting tourists, business people and immigrants. In the past 20 years, the lifestyles of the Italian and Spanish population have changed radically, especially those of women, who have adopted behaviour models traditionally associated with men. Among the most important changes there has been the increasing number of women who smoke, and more recently, who drink, reflecting their changing role in the society [1].

The available data from the most recent Italian National Surveys on use and abuse of alcohol show that the percentage of women of childbearing age who declare daily intake of any alcoholic drink is around 7% between 18 and 44 years of age respectively while that of risky consumers (those who declare to exceed the daily ethanol dose of 20 g) is 6.8 and 4.6% at 18-24 and 25-44 years of age, respectively [1,2]. Concerning Spanish women, the percentage who declare daily intake of any alcoholic drink ranged between 1-2% at 15-34 years of age, increasing to 5.1% at 35-44 years of age [3]. Binge drinking is declared by the 13-15% Spanish women of childbearing age and the rate of excessive Spanish drinkers (excessive drinking was defined as consumption of more than 700 ml of pure ethanol per week) was reported for the 0.2% Spanish women [3,4].

Consequently, it is conceivable that a significant number of women who are problem drinkers and are of child bearing age, will not refrain from ethanol drinking during pregnancy and may give birth to an infant in utero exposed to this toxin. Since prenatal exposure to ethanol has been considered one of the principal diagnostic criteria (or the principal itself) for fetal alcohol syndrome (FAS) and fetal alcohol spectrum disorder (FASD), an early postnatal objective diagnosis by meconium testing for fatty acid ethyl esters (FAEEs) and ethyl glucuronide associated to clinical and neurological follow-up of exposed newborns has been suggested [5-7]. However, universal neonatal screening by meconium testing of alcohol biomarkers is, at moment, too expensive in terms of laboratory facilities and skilled personnel to be performed.

Indeed, there are no Italian or Spanish national data on FAS/FASD prevalence, no national clinical protocols for FAS/FASD diagnosis and finally no national cohort studies. The only Italian experience of a field study, aimed to assess the prevalence of FAS and FASD by retrospective cohort study in a restricted area of Rome province (Lazio region). This study, conducted on 543 children from primary schools showed a striking FAS and FADS prevalence of 0.37 and 2.3% examined children raising the question of whether FASD is more common in the Mediterranean countries than previously estimated [8].

Most of the research on the prevalence of FAS and FASD comes from North America [9,10]; nevertheless, a Canadian study has documented that, due to lack of training, a substantial number of physicians do not feel comfortable in making a diagnosis of FAS [11]. Similarly, an American survey confirmed that pediatricians are knowledgeable about fetal alcohol syndrome but do not feel adequately trained to integrate the management of this diagnosis or prevention efforts into everyday practice [12]. A recent Israeli study [13] corroborated this latter statement demonstrating that those physicians who are at the forefront of diagnosing, treating and preventing FAS and FASD surprisingly declared their limited experience in dealing with the issue in a practical way. Finally, also Australian health professionals dealing with FAS and FASD identified in two subsequent surveys the need for educational materials for themselves and their clients [14,15].

These differences in opinion and inability to diagnosis FAS and FASD may reflect inadequacies in medical training regarding ethanol use and abuse during pregnancy and more specifically about these patterns of mental and physical defects caused by in utero exposure to the toxin.

Since recently an Italian-Spanish joint research group disclosed an alarming prevalence of fetal ethanol exposure in Barcelona, Spain and more recently in Rome and Reggio Emilia, Italy by meconium testing [16,7], the same group sought to evaluate awareness of the diagnosis of FAS and FASD and maternal alcohol use during pregnancy in Italian and Spanish neonatologists and paediatricians.

## Methods

The self-administered questionnaire was e-mailed to the 400 Italian neonatologists members of the Italian Society of Neonatology, SIN, by the Society secretary. The first contact was in June 2010 by e-mail. A second and a third e-mail were sent if no answers were received within a month. Finally, a telephone call was made to the neonatologists, who did not answer even to the third mail.

In order to also evaluate the know-how of paediatricians, the same questionnaire was proposed to participants to a special sessions dedicated to diagnosis of FAS and FASD and maternal ethanol use during pregnancy at the 66^th ^Italian Paediatric Congress (Rome 20-23 October 2010), and during the First Spanish Congress on "Drugs of Abuse and Pregnancy" (Murcia, 15-16 September 2010). Questionnaire was distributed and collected, once completed, by Congress Hostesses before the start of the sessions.

The individual identifiers of the questionnaires were stripped once the surveys had been received and that all data held were treated as being confidential.

The questionnaire was the Italian adapted version of questionnaire submitted to the family physicians by the Canadian investigators on FASD [11], with some of which our Italian-Spanish investigation group has been worked since many years.

It was five-pages long consisting of multiple choice questions. There were sixteen questions that were divided into three sections: 1) epidemiology of the problem, 2) identification of newborns at risk and the ability to diagnose FAS and FASD indicating key factors in the diagnosis making, 3) identification of mothers at risk, or the ability to identify factors related to problem drinking in pregnant women by asking which tools they use for the assessment of ethanol use. This last section was introduced to investigate the ability of professionals to identify factors related to problematic drinking in pregnant women, by asking them which tools they use or suggest together with gynaecologists and nurses for the assessment of maternal ethanol use during pregnancy.

The questionnaire and the study were approved by the Institutional Ethical Committee of Istituto Superiore di Sanità, conducted in accordance with the Declaration of Helsinki and signed informed consent was obtained from all the neonatologists and paediatricians, who completed the questionnaires.

Since this was an exploratory survey of neonatologists and paediatricians awareness of the diagnosis of FAS and FASD and maternal ethanol use during pregnancy, descriptive statistics in numbers and percentages were used to present the results. Confidence interval at 95% (C.I. 95%) for the proportions using the Wald's construction, with an alpha = 0.05 was also calculated.

## Results

A total of 63/400 (16%) Italian neonatologists, 41 Italian and 152 Spanish paediatricians (100% of the participants to the above-reported sessions during national paediatric Congresses) returned the completed questionnaires.

The first three questions asked were: "in Italy/Spain the percentage of pregnant women consuming ethanol 1)any time, 2) daily or 3) problematically is a) unknown because never studied, b) unknown because impossible to estimate, or c) well known (percentage given). Main results are summarised in Additional File 1. With regards the percentage of newborns with presumed FAS or FASD, the majority of Italian neonatologists and paediatricians (over 50% respondents) rated a percentage while the majority of Spanish paediatricians (over 60% respondents) answered "unknown because never studied (Additional File 1).

To assess knowledge regarding FAS and FASD facts in general, responders were asked to give their opinion on the accuracy of six statements they had to evaluate. As a group, the Italian and Spanish respondents agreed that these statements were accurate (Additional File 1).

The respondents were then asked about identification of newborns at risk. According to 54% Spanish paediatricians and 60% Italian neonatologists, the identification of newborns at risk is more accurate analysing neonatal biomarkers. However, few responders (around 2-3%) indicated the correct neonatal biomarkers (e.g FAEEs and ethyl glucuronide in meconium), while the others listed generic parameters (e.g. gamma GT, carbohydrate deficient transferrin, alcoholuria).

While 45% Italian paediatricians considered maternal history (e.g. carbohydrate deficient transferrin, acetaldehyde, transaminases) important to study, 35.7% of them were in favour of neonatal biomarkers.

From a list of FAS associated features, the responders were asked to select the most important features to aid the diagnosis. Main results are summarised in Additional File 1. The responders were next asked to select most important factors in positively influencing the quality of life for a child diagnosed with FAS and FASD from a list which included: actual amount of alcohol taken by the mother during pregnancy, concomitant presence of vertically transmitted infection and finally early diagnosis. About 52% Italian neonatologists, 78% Italian paediatricians and only 4% Spanish paediatricians identified the latter as being the most important factor. In fact the majority of Spanish paediatricians chose the actual amount of alcohol taken by the mother during pregnancy

The final question of this section dealt with the physician's perceived competency at diagnosing FAS or FASD, with 55% of the Spanish physicians feeling that their training was inadequate and 52% of the Italian paediatricians and 64% of the Italian neonatologists feeling that their training was only in part adequate to diagnose FAS and FASD.

The last section of the questionnaire aimed to assess the knowledge of hospital physicians (as referred by the neonatologists and paediatricians) on the assessment of ethanol consumption of pregnant women, women who just delivered and mothers of visited infant. A 59% Italian neonatologists,78.4% Italian paediatricians and 60% Spanish paediatricians reported that they always ask the new mothers about ethanol consumption during pregnancy (Additional File 1). Among the responders who stated that they and their colleagues gynaecologists and nurses always asked news information on the consumption of ethanol during pregnancy, 90% said they had obtained such information through general questions. Just a small minority applied the AUDIT or TWEAK tests widely used in Anglo-Saxon countries as accurate screening methods (Additional File 1). In addition, to identify women at risk, responders judged the maternal clinical history as the key factor followed by maternal and neonatal biomarkers.

To elicit the recommendations responders would most likely give to pregnant women regarding the use of ethanol during pregnancy, they were asked to select statements on amount of ethanol considered safe during pregnancy which they felt were appropriate for counselling. Spanish paediatricians appeared to be the most strict regarding alcohol use during pregnancy, while Italian physicians showed a greater flexibility concerning occasional use of wine and beer (Additional File 1).

## Discussion

Overall, this survey confirmed some of the findings obtained in similar surveys carried out in other geographical areas were the issue of the diagnosis of FAS and FASD and maternal ethanol use during pregnancy was faced in the previous decades [11-15].

This is the first survey concerning FASD awareness among paediatricians and neonatologists in two Mediterranean countries, where ethanol use during pregnancy and consequent prenatal exposure to this toxin has emerged as an overwhelming problem by a series of Italian -Spanish joined investigations [16-21].

One of the important outcomes and a limit of this survey is that very few neonatologists among the ones contacted for the survey completed the questionnaire. In spite of repeated contacts with non-responders to the first approach, probably this survey was not considered as important or neonatologist did not feel comfortable in answering this questionnaire. Email surveys and/or studies carried out in Canada and US [11,12] of health care professionals met similar barriers in recruitment.

Conversely all paediatricians participating in the FAS and ethanol session during the Italian and Spanish Congresses completed the questionnaire but this was a selected population: likely, only interested people attended a specific session on this topic.

Generally speaking, responders either Italian or Spanish based their opinions on percentages of women drinking during pregnancy and on FAS and FASD newborns prevalence on their own perception of the problem since there are no Italian or Spanish data on these two issues. In this concern, even if it has been shown that the majority of health professionals in contact with pregnant women ask for ethanol consumption during pregnancy, this is made with generic questions which can be easily circumvented. It is surprising that only 3.6% of Italian neonatologist and 1.7% of Spanish paediatricians reported using the TWEAK which is currently considered the most accurate screening method [22].

Another surprising result is related to identification of the newborns at risk. Indeed, when diagnostic criteria for FAS were listed as multiple choice, all the Italian and the Spanish professionals were able to correctly identify the principle ones. But then, even though the majority of Italian neonatologists, Spanish pediatricians and one third of Italian pediatricians think biomarkers of neonatal exposure to ethanol as gold standard in identification of newborns at risk, only few of them were able to properly identify them.

It has to be taken in serious account that although over 60% Italian responders and around 80% Spanish responders were aware that ethanol use in pregnancy is dangerous, approximately 50% Italian responders and 40% Spanish ones allowed women to drink sometimes a glass of wine or beer during pregnancy. In addition to that, responders were not updated on current internationally established screening methods, such as AUDIT or better TWEAK tests. It is well known that ethanol crosses the placenta. It can cause problems during pregnancy and can also harm the fetus. Whether or not there is any safe level of ethanol consumption during pregnancy is currently unknown. Nor it is known if any particular stage of pregnancy is the most vulnerable to the effects of drinking [11] . So, in the absence of demonstrated safe limits, total abstinence from ethanol during pregnancy is recommended and should be encouraged [23].

A number of limitations to this study should be mentioned: main one is the relatively small number of physicians surveyed. Unfortunately, many neonatologists did not respond to the survey and this can be a inherent bias, as it is conceivable that the non responders may be less knowledgeable about FAS and FASD and therefore not interested or ashamed in filling the questionnaire. Similarly, all the paediatricians involved in the survey were the most interested in the issue and thus the most informed and the most prone to give the correct answers. Finally, the information would have been more complete if including obstetricians and gynaecologists in the survey, which will be the next step of investigation.

## Conclusion

From the answers obtained to all the questionnaire section it seems that Italian and Spanish physicians feel they need more education about FAS and FASD as they are not confident about being able to make a diagnosis. The same conclusion was reached also by the previously reported similar surveys carried out in Canada, US, Israel and Australia in the recent past [11-15]

Italian and Spanish neonatologists and paediatricians should better understand the importance of asking pregnant women and new mothers about their ethanol use. Therefore they should be trained about screening methods to ensure an accurate record which will allow them to become alerted to women with drinking problem and consequently to their newborn. Although alcohol is the most widely consumed teratogen, most of the education and information in this field is carried out mainly in North America. Despite abundant alcohol consumption in Europe, there is no general politics or intervention on the issue of maternal drinking during pregnancy and consequent prenatal exposure to this toxin.

Since FAS and FASD are one hundred percent preventable, neonatologists and paediatricians from Mediterranean Countries should receive comprehensive education in this field, not only to prevent birth of babies with this syndrome, but also in early detection which can lead to interventions that can improve the quality of life of affected children.

## Authors' contributions

All authors participated in the planning and conception of the questionnaire and the study design. FV and SP were the principal investigators of the study in Italy and OGA and MF in Spain. LM, LT, RS, RP and CM in Italy and OGA and MF were responsible for distribute the questionnaires in Italy and in Spain, respectively. AP was the statistician for analyzing the data. IP drafted the article, and all the authors participated in interpreting the data and critically revising the manuscript fo important intellectual content. All authors read and approved the revised manuscript.

## Competing interests

The authors declare that they have no competing interests.

## Pre-publication history

The pre-publication history for this paper can be accessed here:

http://www.biomedcentral.com/1471-2431/11/51/prepub

## Supplementary Material

Additional file 1 **Tables 1-5**. Click here for file
