# Supplementary material for: A survey of Italian and Spanish neonatologists and paediatricians regarding awareness of the diagnosis of FAS and FASD and maternal ethanol use during pregnancy
Source: BMC Pediatr. 2011 Jun 6;11:51. doi: 10.1186/1471-2431-11-51 (PMC3135544; doi:10.1186/1471-2431-11-51)
Supplement: Additional file 1 — Tables 1-5. [file 1471-2431-11-51-S1.DOC]

Table 1: Awareness of Italian neonatologist and paediatricians and of Spanish paediatricians on drinking **habits** in pregnancy and incidence of FAS FASD in exposed newborns.

**Italian Italian Spanish**

**neonatologists Paediatricians Paediatricians**

**(N=63) (N= 41) (N=152)**

*% pregnant women consuming*

*ethanol in any time is:*

unknown because never studied 52.3 35.0 68.4

**(C.I. 95%) (39.7-64.9) (19.9-50.1) (60.9-75.9)**

unknown because impossible to estimate 12.7 30.0 5.3

**(C.I. 95%) (4.3-21.1) (15.5-44.5) (1.7-8.9)**

well known 35.0 35.0 26.3

**(C.I. 95%) (23-47) (19.9-50.1) (19.2-33.4)**

Percentage given (mean value±SD) 30.7 ±20.7 26.2±26.5 20.4±15.8

*% pregnant women consuming ethanol daily is:*

unknown because never studied 50.8 36.1 68.4

**(C.I. 95%) (38.2-63.4) (20.9-51.3) (60.9-75.9)**

unknown because impossible to estimate 12.7 25.0 5.3

**(C.I. 95%) (4.3-21.1) (11.3-38.7) (1.7-8.9**)

well known 36.5 38.9 26.3

**(C.I. 95%) (24.4-48.6) (23.5-54.3) (19.2-33.4)**

Percentage given (mean value±SD) 18.5±17.9 23.4±19.8 13.9±12.8

*% pregnant women consuming ethanol*

*problematically is:*

unknown because never studied 50.8 14.8 69.0

**(C.I. 95%) (38.2-63.4) (3.6-26) (61.6-76.4)**

unknown because impossible to estimate 7.9 29.6 3.4

**(C.I. 95%) (1.1-14.7) (15.2-44) (0.5-6.3)**

well known 41.3 55.6 27.6

**(C.I. 95%) (28.9-53.7) (39.9-71.3) (20.4-34.8)**

Percentage given (mean value±SD) 5.1±7.8 7.3±5.3 8.7±8.3

*% newborns with FAS is:*

unknown because never studied 15.9 13.9 65.1

**(C.I. 95%) (6.7-25.1) (3-24.8) (57.5-72.7)**

unknown because never studied

through biomarkers 30.2 36.1 12.5

**(C.I. 95%) (18.6-41.8) (20.9-51.3) (7.2-17.8)**

Known 53.9 50.0 22.4

(**C.I. 95%) (41.3-66.5) (34.2-65.8) (15.7-29.1)**

Percentage given (mean value±SD) 1.7±3.4 6.1±14.6 1.7±3.3

*% newborns with FASD is*

unknown because never studied 25.8 25.0 69.6

**(C.I. 95%) (14.8-36.8) (11.3-38.7) (62.2-77)**

unknown because never studied

through biomarkers 35.5 17.9 14.5

**(C.I. 95%) (23.4-47.6) (5.8-30) (8.9-20.1)**

Known 38.7 57.1 15.8

**(C.I. 95%) (26.4-51) (41.5-72.7) (10-21.6)**

Percentage given (mean value±SD) 2.9±5.1 57.1 3.2±2.9 15.8 0.45±1.26

(C.I. 95%= confidence Interval 95%)

Table 2: **Physicians’ opinions/recommendations regarding general knowledge of FAS and FASD (values %)**

Statements **Italian neonatologists(N=63) Italian Paediatricians(N= 41) Spanish Paediatricians(N=152)**

True False Don’t know True False Don’t know True False Don’t know

**Criteria** = FAS is an in identifiable syndrome 96.7 3.3 0.0 89.7 0.0 10.3 98.6 0.0 1.4

**Childhood** = FAS easier to identify during

childhood **than at birth** 19.0 58.6 22.4 12.9 61.5 25.6 7.5 74.1 18.4

Childhood = FASD easier to identify during

childhood **than at birth** 32.1 32.2 35.7 26.4 36.8 36.8 64.1 12.5 23.4

**Dx** = Making early diagnosis

can improve outcome 80.0 12.9 16.1 68.4 26.3 5.3 88.1 5.3 6.6

**Life long** = Dysmorphology is permanent 77.0 8.2 14.8 60.5 13.2 26.3 87.3 3.4 9.3

**Under diagnosis** = FAS is under diagnosed 88.5 1.7 9.8 84.6 2.6 12.8 88.7 0.7 10.6

Table 3 **Identification of FAS diagnostic criteria***

FAS Features **Italian Italian Spanish**

**neonatologists Paediatricians Paediatricians**

**(N=63) (N= 41) (N=152)**

Growth retardation 92.0% 87.8% 85.5%

**(C.I. 95%) (85.2-98.8) (77.5-98.1) (79.9-91.1)**

CNS neurodevelopmental

abnormalities 58.7% **41.6% 63.8%**

**(C.I. 95%) (46.3-71.1) (26-57.2) (56.1-71.5)**

Facial dysmorphology **76.1% 56.0% 89.5%**

**(C.I. 95%) (65.4-86.8) (40.3-71.7) (84.6-94.4)**

Behavioural problems 65.0% 58.5% 85.5%

**(C.I. 95%) (53-77) (42.9-74.1) (79.9-91.1)**

Cardiac malformations 11.1% 34.1% 7.2%

**(C.I. 95%) (3.2-19) (19.9-49.1) (3.1-11.3)**

Mental retardation 63.5% 39.0% 84.2%

**(C.I. 95%) (51.4-75.6) (23.6-54.4) (78.4-90)**

Confirmed maternal

alcohol exposure 57.1% 68.3% 81.5%

**(C.I. 95%) (44.6-69.6) (53.6-83) (75.3-87.7)**

*The physicians were asked to rate the most important features of FA (more than one option could be selected)

**(C.I. 95%= confidence Interval 95%)**

Table 4 **Identification of pregnant women at risk of excessive drinking habit***

Actions **Italian Italian Spanish**

**neonatologists Paediatricians Paediatricians**

**(N=63) (N= 41) (N=152)**

*Daily practice: ask to the mother about her alcohol consumption in pregnancy (%)*

Always 59.7 60.0 78.4

**(C.I. 95%) (47.3-72.1) (44.5-75.5) (71.8-85)**

Never 12.9 5.7 6.9

**(C.I. 95%) (4.5-21.3) (0-13) (2.8-11)**

Sometimes 27.4 34.3 14.7

**(C.I. 95%) (16.2-38.6) (19.3-49.3) (9-20.4)**

*Medical register and information about alcohol consumption(%)*

**No information** 38.7 **61.5** 74.7

**(C.I. 95%) (26.4-51) (46.1-76.9) (67.7-81.7)**

Information are present: 61.3 38.5 25.3

**(C.I. 95%) (49-73.6) (23.1-53.9) (18.3-32.3)**

a)in the form of questions 96.4 92.3 98.3

**(C.I. 95%) (91.7-100) (83.9-100) (96.2-100)**

b)in the form of test

(AUDIT, TWEAK) 3.6 7.7 1.7

**(C.I. 95%) (0-8.3) (0-16.1) (0-3.8)**

*Identification of women at risk*

Clinical history 29.7 32.6 19.7

**(C.I. 95%) (18.2-41.2) (17.8-47.7) (13.3-26.1)**

Questionnaire self-report 5.5 6.1 3.8

**(C.I. 95%) (0-11.2) (0-13.7) (0.7-6.9)**

Maternal biomarkers 13.5 8.2 9.3

**(C.I. 95%) (4.9-22.1) (0-16.9) (4.6-14)**

Newborn biomarkers 9.5 6.1 6.5

**(C.I. 95%) (2.1-16.9) (0-13.7) (2.5-10.5)**

None of the above 0.0 0.0 0.0

All of the above 41.8 47.0 60.7

**(C.I. 95%) (29.4-54.2) (31.2-62.8) (52.9-68.5)**

*(only one option could be selected)

**(C.I. 95%= confidence Interval 95%)**

Table 5 **Physicians recommendations regarding alcohol use during pregnancy***

Recommendation **Italian Italian Spanish**

**neonatologists Paediatricians Paediatricians**

**(N=63) (N= 41) (N=152)**

The amount of alcohol considered

safe for the fetus is unknown 38.0% 59.5% 74.3%

**(C.I. 95%) (25.8-50.2) (44-75) (67.3-81.3)**

No alcohol is recommended

throughout pregnancy 66.1% 63.2% 86.0%

**(C.I. 95%) (54.2-78) (48-78.4) (80.4-91.6)**

No alcohol is recommended

in the first trimester 69.3% 71.1% 87.4%

**(C.I. 95%) (57.7-80.9) (56.8-85.4) (82.1-92.7)**

A glass of wine or beer occasionally

is not likely to be of concern 56.4% 51.4% 37.6%

**(C.I. 95%) (43.9-68.9) (35.6-67.2) (29.8-45.4)**

Other

(indicates options not listed above) none none none

*(more than one option could be selected)

**(C.I. 95%= confidence Interval 95%)**
